# Novel Subtypes of Pulmonary Emphysema Based on Spatially-Informed Lung Texture Learning: The Multi-Ethnic Study of Atherosclerosis (MESA) COPD Study

**DOI:** 10.1109/TMI.2021.3094660

**Published:** 2021-11-30

**Authors:** Jie Yang, Elsa D. Angelini, Pallavi P. Balte, Eric A. Hoffman, John H. M. Austin, Benjamin M. Smith, R. Graham Barr, Andrew F. Laine

**Affiliations:** Department of Biomedical Engineering, Columbia University, New York, NY 10027 USA; Department of Biomedical Engineering, Columbia University, New York, NY 10027 USA, also with the ITMAT Data Science Group, NIHR Imperial BRC, London W2 1NY, U.K., and also with the Department of Metabolism-Digestion-Reproduction, Imperial College, London SW7 2BX, U.K.; Department of Medicine, Columbia University Medical Center, New York, NY 10032 USA; Department of Radiology, the Department of Medicine, and the Department of Biomedical Engineering, The University of Iowa, Iowa City, IA 52242 USA; Department of Radiology, Columbia University Medical Center, New York, NY 10032 USA; Department of Medicine, Columbia University Medical Center, New York, NY 10032 USA, and also with the Department of Medicine, McGill University Health Center, Montreal, QC H4A 3J1, Canada; Department of Medicine and Epidemiology, Columbia University Medical Center, New York, NY 10032 USA; Department of Biomedical Engineering, Columbia University, New York, NY 10027 USA

**Keywords:** Lung CT, emphysema, unsupervised learning, spatial mapping, lung texture

## Abstract

Pulmonary emphysema overlaps considerably with chronic obstructive pulmonary disease (COPD), and is traditionally subcategorized into three subtypes previously identified on autopsy. Unsupervised learning of emphysema subtypes on computed tomography (CT) opens the way to new definitions of emphysema subtypes and eliminates the need of thorough manual labeling. However, CT-based emphysema subtypes have been limited to texture-based patterns without considering spatial location. In this work, we introduce a standardized spatial mapping of the lung for quantitative study of lung texture location and propose a novel framework for combining spatial and texture information to discover spatially-informed lung texture patterns (sLTPs) that represent novel emphysema subtype candidates. Exploiting two cohorts of full-lung CT scans from the MESA COPD (n = 317) and EMCAP (n = 22) studies, we first show that our spatial mapping enables population-wide study of emphysema spatial location. We then evaluate the characteristics of the sLTPs discovered on MESA COPD, and show that they are reproducible, able to encode standard emphysema subtypes, and associated with physiological symptoms.

## I. Introduction

PULMONARY emphysema is morphologically defined by the enlargement of airspaces with destruction of alveolar walls distal to the terminal bronchioles [1]. Emphysema overlaps considerably with chronic obstructive pulmonary disease (COPD), which is the third leading cause of death in the world [2]. Based on small autopsy series, pulmonary emphysema is traditionally subcategorized into three standard subtypes, which can be visually assessed on computed tomography (CT) of the lung, using the following definitions:

- *Centrilobular emphysema* (CLE): low-attenuation regions surrounded by normal lung, and located centrally in the secondary pulmonary lobules [3]. Classically, its distribution is predominantly in the apical regions of the lungs;
- *Panlobular emphysema* (PLE): low-attenuation regions which are uniformly diffuse in the secondary pulmonary lobules [4]. Classically, its distribution is predominantly in the basal regions of the lungs;
- *Paraseptal emphysema* (PSE): low-attenuation regions adjacent to pleura and to intact interlobular septa, typically found in juxtapleural lobules adjacent to mediastinal and costal pleura [3]. Classically, its distribution is predominantly in the upper and middle lung zones.

The three standard emphysema subtypes are associated with distinct risk factors and clinical manifestations [5], and may represent different diseases. However, given that these subtypes were initially defined at autopsy before the availability of CT scanning, there have been disagreements among pathologists on the very existence of such pure subtypes [6], current guidelines modify them [3] and a large emphysema study on 1,800 autopsies in [7] ignored them completely, mainly for practical reasons. Radiologists’ interpretation of these subtypes on CT scans is labor-intensive, with substantial intra- and inter-rater variability [3], [4], [8].

Automated CT-based analysis enables *in vivo* study of emphysema patterns, and has received increasing interest recently [9], [10], either via supervised learning for replicating emphysema subtype labeling as in [11]–[15], or via unsupervised learning for the discovery of new emphysema subtypes as in [16]–[18].

Preliminary CT-based clinical studies suggest that regional analysis will be instrumental in advancing the understanding of multiple pulmonary diseases [19]. In the case of emphysema, it is suspected that different emphysema subtypes affect the lungs in specific anatomical regions. But the problem of how many subtypes exist, how they evolve in time and how they vary with spatial (anatomical) location is still unsolved. To date, categorization of emphysema on CT images has relied only on analysis of local textural patterns, using either grey-level co-occurrence matrix (GLCM) features [12], [16], texton features [13], [14], or local binary pattern (LBP) features [11]. All these approaches use intensity information without consideration of spatial location.

In two previous studies [17], [18], we proposed to use local textural patterns to generate unsupervised lung texture patterns (LTPs) followed by LTP-grouping based on their spatial co-occurrence in local neighborhoods. Such separate use of intensity and spatial information cannot guarantee spatial and textural homogeneity of the final LTPs.

In this study, we propose to perform discovery of LTPs via unsupervised clustering of joint spatial and textural information of local texture patterns. Spatial information can be inferred from crude partitioning of the lung with subdivisions of Cartesian coordinates or by segmenting the lung into zones (e.g. upper, lower) [4] or lobes [20]. However, such approaches have limited spatial precision and lack relative information such as peripheral versus central positioning, which is important in defining paraseptal emphysema and subpleural bullae. We introduced in [21] a new standardized lung shape spatial mapping, called Poisson distance conformal mapping (PDCM), which enables detailed, precise and standardized mapping of voxel positions with respect to the lung surfaces. This paper further refines the PDCM algorithm and exploits it for the study of emphysema spatial patterns across populations of CLE-, PLE- and PSE-predominant subjects. This paper also provides an exhaustive description of the framework for combining spatial and texture information in the unsupervised discovery of *emphysema-specific* texture patterns, which are called spatially-informed LTPs (sLTPs).

Exploiting a cohort of 317 full-lung CT scans from the MESA COPD study [4], and 22 longitudinal CT scans from the EMCAP study [22], the discovered sLTPs are extensively evaluated as emphysema subtype candidates in terms of reproducibility with respect to training sets, labeling task and scanner generations, ability to encode standard emphysema subtypes, and associations with respiratory symptoms. A graphical pipeline of the learning and evaluation steps is provided in the Supplementary Material.

## II. Method

### A. Overview

The proposed unsupervised learning framework is structured in four main steps to model the spatial and texture features within emphysema-like lung, and generate the sLTPs emphysema subtype candidates:

1. Generate spatial mapping of the lung masks: mapping voxels within the lung masks into a custom Poisson distance map (PDM) to encode the “peel to core” distance, and a conformal mapping (PDCM) to distinguish superior versus inferior, anterior versus posterior and medial versus lateral voxel positions;
2. Encode regions of interest (ROIs) within emphysema-like lung: sampling ROIs from emphysema segmentation masks, and generating spatial features (based on spatial mapping) and texture features of each ROI;
3. Discover an initial set of LTPs: clustering training ROIs into a large number of clusters, based on texture features, and then iteratively augment the LTPs with spatial information via regularization;
4. Generate the final set of sLTPs: measure the similarity between LTPs in the initial set, group similar / redundant LTPs and generate the final set of sLTPs via partitioning a similarity graph.

We now detail these four steps individually.

### B. Spatial Mapping of the Lung Masks

To generate spatial mapping of the lung masks, we first use the concept of Poisson distance map (PDM), introduced in [23], to encode the shape of individual lung masks *V*. PDM is commonly used for characterizing the silhouette of an object via continuous labeling of voxel positions with scalar field values *U*_3*d*_ in the range of [0, 1]. In our case, the field value *U*_3*d*_ encodes the “peel to core” distance between a given voxel and the external lung surface *∂V*. This field is computed by solving the following Poisson equation:

(1) $$\;\;\text{Δ}U_{3d}\left(x,y,z\right)=-1,\;\text{for}\;\left(x,y,z\right)\in V\;\text{subject to}\;U_{3d}\left(x,y,z\right)=0,\;\text{for}\;\left(x,y,z\right)\in \partial V$$

where $\text{Δ}U=U_{xx}+U_{yy}+U_{zz}$ is the Laplacian operator based on 2nd-order spatial derivatives along *x, y, z*.

The solution for *U* proposed in [23] is guaranteed to be smooth according to [24]. It has the advantage of generating distance values that are sensitive to global shape characteristics, unlike other distance metrics (e.g. Euclidian or Metropolis distances) which exploit single contour points. PDM can therefore reflect rich shape properties of the lung.

**Fig. 1.**
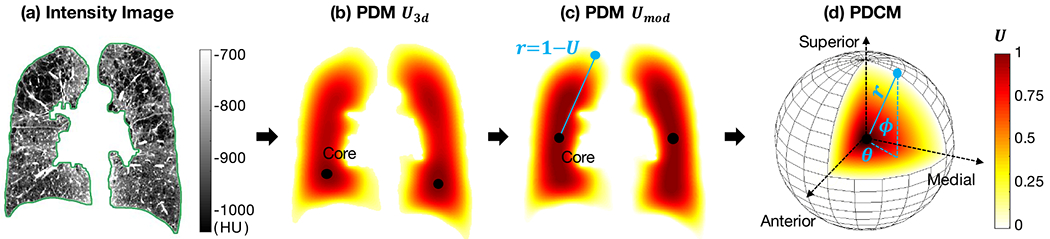
Illustration of the lung shape spatial mapping: **(a)** Original intensity image (visualized on a coronal slice, with the green contour indicating the boundary of lung mask); **(b)** Corresponding Poisson distance map (PDM) *U*_3d_ with values in range [0, 1] that measure the “peel to core” 3D distance to the lung mask external surface; **(c)** Modified PDM *U*_mod_ for comparable core locations between subjects; **(d)** 3D conformal mapping of the lung PDM to a sphere leading to a Poisson distance conformal map (PDCM) where pixels are assigned three coordinate values (*r, θ, ϕ*) which enable to distinguish superior vs. inferior, anterior vs. posterior and medial vs. lateral positions, in addition to “peel to core” distance.

The core of the PDM is the set of voxels (one or very few) where *U*_3*d*_ (*x, y, z*) = 1. The PDM generated from a lung surface generally exhibits nice star-shaped profiles when viewed in axial cuts, with maxima near the center. On the other hand, core positions can vary greatly among subjects along the superior-inferior axis, due to variable morphologies of the lungs, especially near the heart and at the base. We illustrate an example in Fig. 1 (b) where the PDM generated with Equation (II-B) has core point(s) located close to the base of the lung rather than concentrated toward the middle of the longitudinal axis. We propose the following calibration of lung PDMs to (1) prevent *U*_3*d*_ = 1 in most apical and basal regions, and (2) enforce *U*_3*d*_ = 1 in a large range of axial slices. This makes PDM values numerically more consistent between subjects over a comparable range of axial slices.

We denote $U_{3d}^{max}\left(i\right)$ the maximal in-slice value of *U*_3*d*_(.,.,*i*), where the *i*^*th*^ axial slice index is counted from the apex. We denote *i*_*v*_% the highest slice index value such that the total lung volume sumed over all slices with lower indices is < *V*% of the total lung volume. A normalized version (denoted as *U*_2*d*_) of the original PDM *U*_3*d*_, is then defined, per axial slice index *i*, as $U_{2d}\left(.,\;.,i\right)=U_{3d}\left(.,\;.,i\right)/U_{3d}^{max}\left(i\right).$ We further define *U*_*mod*_ by combining *U*_3*d*_ and *U*_2*d*_ values, as follows:

(2) $$U_{mod}(.,.,i)=U_{2d}(.,.,i),\;\forall i_{u}⩽i⩽i_{d}\;U_{mod}(.,.,i)=U_{3d}(.,.,i)/U_{3d}^{max}\left(i_{u}\right),\;\forall i<i_{u}\;U_{mod}(.,.,i)=U_{3d}(.,.,i)/U_{3d}^{max}\left(i_{d}\right),\;\forall i>i_{d}$$

with *i*_*u*_ (resp. *i_d_*) the smallest (resp. highest) slice index where $U_{3d}^{max}$ reaches a local maximum. To ensure that a consistent portion of the lung is included in [*i_u_*, *i_d_*] we further enforce: if *i_u_ > i*_25%_ then *i_u_ = i*_25%_ (resp. if *i_d_ < i*_75%_ then *i_d_ = i*_75%_). We illustrate in Fig. 1(c) an example where *U*_*mod*_ = 1 over a large range of axial slice indices and exhibits decreasing values when moving toward the apex or the base of the lung.

To equip the PDM with a coordinate system, we set the final core coordinate center point as the point on the axial slice index *i*_50%_ where *U*_*mod*_(*x, y, i*_50%_) = 1 and closest to the 2D center of mass of the axial lung mask (in case of multiple candidates, we would select one abitrarily, but such situation was not encountered on our dataset.).

To uniquely encode 3D voxel positions, we define radial values *r* = 1 − *U*_*mod*_ and add conformal mapping of voxels positions onto a sphere, generating a Poisson distance conformal map (PDCM). We encode superior versus inferior, anterior versus posterior and medial versus lateral voxel positioning via latitude and longitude angles (*θ, ϕ*) with respect to the PDM core defined above and standard image axis. The generation of the spatial PDCM mapping is illustrated in Fig. 1(d).

The PDCM spatial mapping will be exploited for sLTP learning, and also to study population-based spatial location of emphysema, as reported in Section III-B.

### C. Texture and Spatial Features

#### 1) Prior Emphysema Segmentation and ROI Sampling:

Texture and spatial analysis is performed within local ROIs centered on a subset of lung voxels. Sampling ROIs from emphysema-like lung requires prior emphysema segmentation. In this study, we exploited a training cohort of full-lung CT scans and their associated emphysema masks, which are generated using both a thresholding-based voxel selection and a hidden Markov measure field (HMMF) segmentation [25]. For thresholding, voxels with attenuation below −950 HU are selected. The HMMF segmentation enforces spatial coherence of the labeled emphysematous regions, and relies on parametric modeling of intensity distributions within emphysematous and normal lung tissues to adapt to individual and scanner variability. Percent emphysema measures the proportion of emphysematous voxels within the lung region, and is denoted *%emph*_−950_ or *%emph*_HMMF_, depending on the emphysema segmentation method.

In preliminary implementations, we tested several options for ROI sampling such as keypoint sampling in [17] and regular sampling in [18]. In this study, we use the systematic uniform random sampling (SURS) strategy as suggested in [26] for use on lung CT scans. Each individual lung mask is randomly sampled via dividing the bounding box of the lung into 3D stacks, and then selecting voxels per stack with a random shift of positions. Two parameters are used for the sampling: *β*_1_ is used for the random shift of positions and *β*_2_ is used to set the number of sampled voxels per stack. The SURS sampling ensures even representation of all lung regions while introducing variability in the position of sampled points with the random shift parameter *β*_1_. Only ROIs with both percent emphysema *%emph*_−950_ > 1% and *%emph*_HMMF_ > 1% are retained for training to ensure sufficient representation of emphysematous regions (i.e. each training ROI has a minimal proportion of emphysema but can be a mixture of normal and emphysematous tissues).

#### 2) Texture Features:

We use texton-based texture features to characterize each ROI, which model textures as the repetition of a few basic primitives (called textons), and were shown to outperform other texture features in unsupervised lung texture learning in [18]. A texton codebook is constructed by retaining the cluster centers (textons) of raw pixel representations of small-sized training patches. The clustering is performed with *K*-means. By projecting all small-sized patches of a ROI onto the codebook, the texton-based feature of the ROI is the normalized histogram of texton frequencies.

#### 3) Spatial Features:

To generate spatial features of individual ROIs, we divide the lung masks into lung sub-regions by discretizing our continuous lung shape spatial mapping with a minimal granularity. We divided *r* ∈ [0, 1] into 3 regular intervals to distinguish pleural from mid from core regions, divided *θ* ∈ [0, 2*π*] into 4 regular intervals to distinguish anterior, medial, posterior and lateral regions, and divided *ϕ* ∈ [−*π*/2, *π*/2] into 3 regular intervals to distinguish inferior, mid-level and superior regions. The spatial feature of each ROI is a one-hot vector indicating the lung sub-region it belongs to. Ordering of the bins that represent the sub-regions is done via arbitrary spatial rastering as no assumption needs to be made on spatial adjacency of adjacent bins.

### D. Initial Augmented LTPs

We formulate the discovery of spatially-informed lung texture patterns (sLTPs) as an unsupervised clustering problem. One key factor in unsupervised clustering is the choice of the number of clusters. The algorithm is expected to find finer-grained emphysema subtype candidates than the three standard ones. Therefore, the number of clusters should be large enough to handle the diversity of textures encountered in the lung volumes (i.e. good intra-cluster homogeneity), and small enough to avoid redundancy (i.e. good inter-cluster differences) for clinical interpretation. Fixing *a priori* the number of clusters may prevent the discovery of rare patterns. We therefore propose a two-stage learning strategy, where we first generate an arbitrary large number of fine-grained lung texture patterns (LTPs), and then group similar LTPs to produce the final set of sLTPs, according to a dedicated metric.

LTPs {*LTP_k_*} ({·} denotes a set of variables hereafter) are characterized by their texture and spatial feature centroids $\left({\bar{FT}}_{LT\;P_{k}},{\bar{FS}}_{LT\;P_{k}}\right),$ which are encoded as histograms via averaging over assigned ROIs. An initial set of LTPs is generated by clustering with *texture* features, and is then augmented with *spatial* regularizations via iterative updates of the centroids and the ROIs assignements as described in Algorithm 1 and using the following mixed *χ*^2^-*ℓ*^2^ similarity metric to enforce spatial concentration of LTPs while preserving their intra-class textural homogeneity:

(3) $${\left\{{\text{Λ}}_{LT\;P_{k}}^{\left(t\right)}\right\}}_{\left\{\lambda ,W,\gamma \right\}}^{*}=\underset{\left\{{\text{Λ}}_{LT\;P_{k}}^{\left(t\right)}\right\}}{\text{argmin}}\sum_{k}\sum_{x\in {\text{Λ}}_{LT\;P_{k}}^{\left(t\right)}}\chi^{2}\left(FT_{x},{\bar{FT}}_{LT\;P_{k}}^{\left(t-1\right)}\right)\;\;\;\;\;\;\;\;\;+\lambda \cdot W\cdot {\left\|FS_{x}-{\bar{FS}}_{LT\;P_{k}}^{\left(t-1\right)}\right\|}_{2}^{2}\;\;\;\;\;\;\;\;\;+\gamma \cdot 1[\chi^{2}\left(FT_{x},{\bar{FT}}_{LT\;P_{k}}^{\left(t-1\right)}\right)\;\;\;\;\;\;\;\;\;\;\;>\;\underset{x^{\prime }\in {\text{Λ}}_{LT\;P_{k}}^{(t-1})}{P_{95}}\left[\chi^{2}\left(FT_{x^{\prime }},{\bar{FT}}_{LT\;P_{k}}^{\left(t-1\right)}\right)\right]]$$

where *P*_95_ denotes the 95^*th*^ percentile, ${\text{Λ}}_{LT\;P_{k}}^{\left(t\right)}$ denotes the set of ROIs that are labeled as *LTP*_*k*_ at iteration *t* and ${\left\{\wedge_{LT\;P_{k}}^{\left(t\right)}\right\}}_{\left\{\lambda ,W,\gamma \right\}}^{*}$ denotes the optimal labeling identified with a set of parameters {*λ, W, γ*} and the centroids updated at iteration *t* − 1. Designing proper distance metrics for histograms plays a crucial role in many computer vision tasks. Two popular choices are the *χ*^2^ and the *ℓ*^2^ distance metrics. The latter equally weights distances of all bins and is favored to compare one-hot vectors, while the former is a weighted distance favored to compare probability distributions. For the texture feature histograms that encode distributions over textons the first distance metric *χ*^2^ (·) measures the *χ*^2^ distance between the textural features of a ROI *x* and the centroid of *LTP*_*k*_. For the spatial features that are sparse one-hot vectors for individual ROIs, the second distance metric ${\left\|\cdot \right\|}_{2}^{2}$ measures the *ℓ*^2^ distance between the spatial features of a ROI *x* and the centroid of *LT P*_*k*_. A textural penalty term is then introduced as the third term, where 1 is the indicator function. Update of LTP centroids (step 2 in Algorithm 1) is performed after relabeling each ROI with the LTP to which it has the smallest weighted feature distance without turning on the textural penalty.


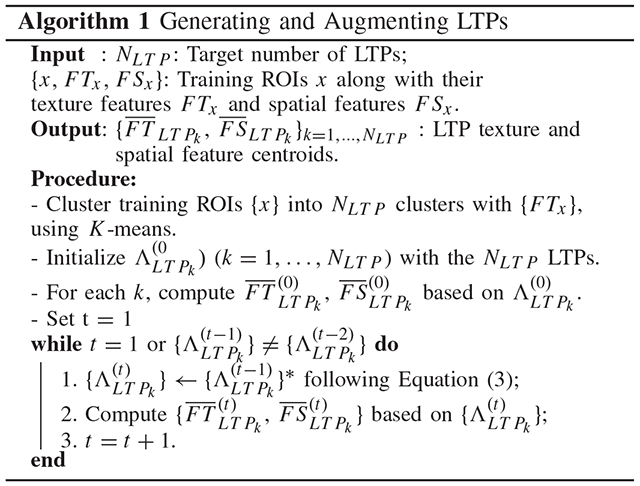


#### Parameter W:

This parameter is used to scale contributions between textural and spatial feature distances so that *λ* can be tuned within a small range of values. We defined it as:

(4) $$W=\frac{SST_{T}}{SST_{S}}=\frac{\sum_{x}\chi^{2}\left(FT_{x},\sum_{x}FT_{x}/N\right)}{\sum_{x}{\left\|FS_{x}-\sum_{x}FS_{x}/N\right\|}_{2}^{2}}$$

where *SST*_*T*_ and *SST*_*S*_ are respectively the texture and spatial *total* sum-of-square distances, computed on the whole *N* training ROIs to measure the overall diversity of texture and spatial features.

#### Parameter λ:

This parameter controls the spatial regularization which will inevitably decrease textural homogeneity of individual LTPs. The value of *λ* is set as follows. First we define *SSW*_*T*_ as the initial sum-of-square *intra-cluster* homogeneity of texture features without spatial regularization:

(5) $$SSW_{T}=\sum_{k}\sum_{x\in {\text{Λ}}_{LT\;P_{k}}^{(0})}\chi^{2}\left(FT_{x},{\bar{FT}}_{LT\;P_{k}}^{\left(0\right)}\right)$$

Then we define $SSW_{T}^{\lambda }$ as the *SSW*_*T*_ measured on augmented LTPs with spatial regularization enforced with *λ* ∈ [0, 2]. Final value of *λ* is set to:

(6) $$\;\lambda^{*}=\mathrm{argmax}\;\lambda \;\text{s.t.}\;\text{Δ}SSW_{T}(\lambda )<L_{T}\;\text{where}\;\text{Δ}SSW_{T}(\lambda )=\frac{SSW_{T}^{\lambda }-SSW_{T}}{SSW_{T}}\%$$

In the context of unsupervised discovery, we hereby spatially regularize the augmented LTPs via an empirically acceptable textural homogeneity loss with the threshold *L*_*T*_ (set based on data observations, as reported in Section III).

#### Parameter γ:

This parameter weights the textural penalty term which is used for ROI labeling. We set *γ* = ∞ to prevent a ROI from being labeled to a spatially preferred but texturally dissimilar LTP.

### E. Final sLTPs

In this final step, we generate sLTPs by partitioning a weighted undirected graph *G* where nodes are the *N*_*LT P*_ initial augmented LTPs. As in [21], we define the edge weight between nodes *i* and *j* as the average replacement ratio of training ROIs relabeled from label *LT P_i_* to *LT P_j_* if *LT P_i_* is removed from the set of centroids and vice versa. In the replacement task, a ROI with a textural distance to the *LT P*_*k*_ centroid exceeding the maximal intra-cluster textural distance of *LT P*_*k*_ is not re-labeled. To prevent weak associations of LTPs that are not easily replaceable, we remove edges with weights lower than 0.5 (i.e. 50% replacement). Indeed, graph partitioning tends to preserve nodes that are not connected, which in our case would correspond to LTPs that are not easily replaced by other ones in the labeling task, hence not redundant. We use the Infomap algorithm [27] to partition the similarity graph *G*. As part of its optimization process that minimizes the description length of the network, Infomap selects an optimal number of clusters of aggregated *LT P*_*k*_ which define our final sLTPs. Final texture and spatial centroids of the sLTPs are then computed utilizing the training ROIs labeled in our final {*LT P*_*k*_}.

### F. Labeling of CT Scans With sLTPs

In the test stage, scans in the whole dataset are labeled by extracting sample points and their ROIs {*x*}. Since it is computationally prohibitive to evaluate the textural and spatial features on every voxels within the lung masks, we only label centers of ROIs densely sampled using again SURS. Sampled ROIs with *%emph*_−950_ ⩽ 1% or *%emph*_HMMF_ ⩽ 1% have their center labeled as no-emphysema class. Remaining sampled centers get a sLTP label, via minimization of the following cost metric:

(7) $$\chi^{2}\left(FT_{x},{\bar{FT}}_{sLT\;P_{k}}\right)+\lambda \cdot W\cdot {\left\|FS_{x}-{\bar{FS}}_{sLT\;P_{k}}\right\|}_{2}^{2}$$

Non-sampled voxels are labeled with the sLTP index of the nearest sampled center point via nearest neighbor search within the lung mask (i.e. using a Voronoi diagram). Labeling lung scans with the discovered sLTPs generates histograms of sLTPs, which are efficient lung texture signatures exploited for several tasks, as described in the evaluation sections.

### G. Visualization of the sLTPs Spatial Density

To study the spatial distribution of sLTPs, we generate spatial visualization by scatter plotting of voxels labeled with individual sLTPs in sagittal projections, as follows.

We first randomly sample an initial set of ROIs over each lung via SURS sampling. Each ROI is associated with its center point coordinates (*r, θ, ϕ*) in the PDCMs. To avoid artificial higher densities on the scatter plot in regions close to the core, we adapt the number of ROIs selected per radial regions. The *r* values are binned into *N*_*r*_ intervals with midpoint values *r*_1_, … , *r*_*N_r_*_ to generate isovolumetric subvolumes of the lung. We then define the sub-sampling ratio *α_i_* = *r_i_*/*r*_*N_r_*_ (which approximates the ratio of areas in the scatter plot) and set the number of ROIs sampled per *r* bin to $N_{\mathrm{Iso}V_{i}}=\alpha_{i}\cdot N_{\bar{\mathrm{Iso}V}}$ where $N_{\bar{\mathrm{Iso}V}}$ is a pre-set number of ROIs sampled in the outermost part of the lung.

All ROI centers in the sub-sampled set are converted to (*x, y, z*) Cartesian image coordinates and accumulated in a sagittal single plane, by setting *x* = 0. Final density plots of sLTPs are shown in projected radial coordinates $r^{\prime }=\sqrt{y^{2}+z^{2}}$ and $\phi^{\prime }=atan\left(z/y\right).$ We color code each point on the sagittal projection with the following density measure:

(8) $${Den}_{sLT\;P_{k}}^{\left(r^{\prime },\phi^{\prime }\right)}=\frac{\left|{\text{Λ}}_{sLT\;P_{k}}\cap^{\text{​}}{\text{Λ}}_{\left(r^{\prime },\phi^{\prime }\right)}\right|}{\left|{\text{Λ}}_{sLT\;P_{k}}\right|}/\frac{\sum_{i}\left|{\text{Λ}}_{sLT\;P_{i}}\cap^{\text{​}}{\text{Λ}}_{\left(r^{\prime },\phi^{\prime }\right)}\right|}{\sum_{i}\left|{\text{Λ}}_{sLT\;P_{i}}\right|}$$

where ${\text{Λ}}_{\left(r^{\prime },\phi^{\prime }\right)}$ denotes the set of ROIs at (*r′, ϕ′*) positions. The numerator (first term) in Equation (8) measures the probability of *sLT P*_*k*_ at projected position (*r′, ϕ′*), and the denominator (second term) measures the observed overall probability of (*r′, ϕ′*) to host any *sLT P_i_*.

## III. Experiments & Results

### A. Data

The data used for evaluation consists of full-lung CT scans of 317 subjects. All subjects had underwent CT scanning in the MESA COPD study [4], between 2009–2011. In addition, 22 out of the 317 subjects underwent CT scanning in the EMCAP study [22], between 2008–2009.

For the MESA COPD study, all CT scans were acquired at full inspiration with either a Siemens 64-slice scanner or a GE 64-slice scanner, at 120 kVp, speed 0.5 s, and current (mA) set according to body mass index following the SPIROMICS protocol [28]. Images were reconstructed using B35/Standard kernels with axial pixel resolutions within the range [0.58, 0.88] mm, and 0.625 mm slice thickness.

For the EMCAP study, scans were acquired with a Siemens 16-slice scanner, at 120 kVp, speed 0.5 s, and a current between 169 mA and 253 mA. Images were reconstructed using the B31f kernel with axial resolutions within the range [0.49, 0.87] mm, and 0.75 mm slice thickness.

Emphysema subtypes and severity have previously been assessed visually in the MESA COPD study (details available in [4]). The raters included four experienced chest radiologists from two academic medical centers. They assessed emphysema subtypes on CT scans by assigning a percentage of the lung volume affected by CLE, PLE and PSE respectively. Based on [4], *N* = 205 subjects do not exhibit emphysema, and are used here as the control set of no emphysema (NE) subjects. The remaining *N* = 112 subjects exhibit light (*N* = 53) or mild-to-severe (*N* = 59) emphysema. For these subjects, predominant emphysema subtype is defined as the subtype affecting the greatest proportion of the lungs. In the mild-to-severe cases, there are *N* = 37 CLE-predominant, *N* = 12 PLE-predominant, and *N* = 10 PSE-predominant subjects. Overall population prevalence of emphysema in the MESA COPD cohort is 27%, composed of 14% of CLE-subtype, 9% of PSE-subtype, and 4% PLE-subtype.

In addition, the following clinical characteristics are available for the scans in MESA COPD study (details in [4]): demographic factors (age, race, gender, height, weight); forced expiratory volume in 1 second (FEV1); MRC dyspnea scale measure (5-level scale); six-minute walking test (6MWT) total distance; pre (baseline) 6MWT pulse oximetry; post 6MWT pulse oximetry; reported post 6MWT fatigue; and reported post 6MWT breathlessness. We used these measures for evaluating the clinical significance of the discovered sLTP.

### B. Population Evaluation of Emphysema Using PDCM

**Fig. 2.**
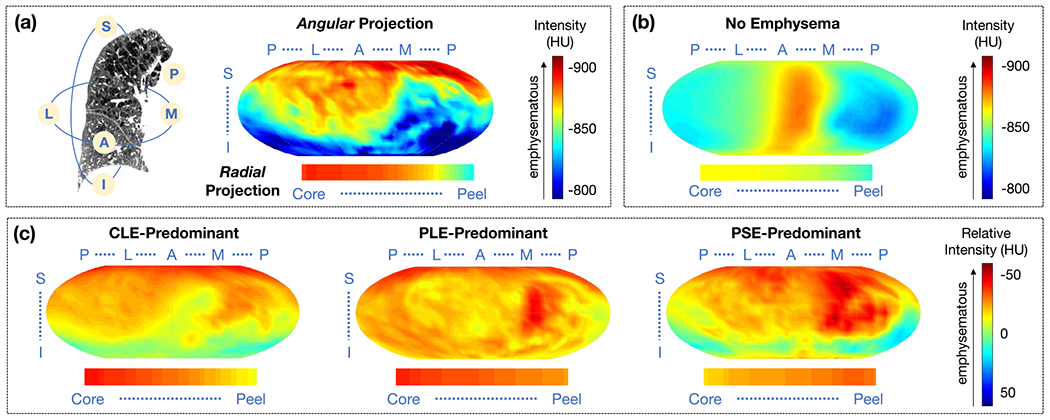
Population evaluation of emphysema using PDCM. **(a)** Illustration of superior (S), inferior (I), medial (M), lateral (L), posterior **(P)** and anterior (A) positions, and PDCM-based intensity projections on a sample right lung. **(b)** Average intensity (in HU) on PDCM-based angular and radial projections for MESA-COPD subjects with no emphysema (N = 205); **(c)** Average relative intensity differences, with respect to **(b)**, on PDCM-based projections for MESA-COPD subjects with CLE-, PLE- and PSE-predominant emphysema (N = 37, 12 and 10 respectively).

We first demonstrate the ability of our proposed PDCM lung shape mapping to study the spatial patterns of emphysema over a population of subjects (cf. Fig. 2). For each scan in MESA COPD study, PDCM maps of voxels inside individual lungs are generated, attributing to each voxel a coordinate (*r, θ, ϕ*). Voxel intensity values in PDCM maps are then averaged and visualized along two types of projections:

1. *Angular projections:* intensity values averaged along *r* for each pair of angular directions (*θ, ϕ*);
2. *Radial projections:* intensity values averaged over all angular directions at a subset of *N*_*r*_ = 60 regular radial positions *r*_1_, … , *r*_*N_r_*_.

An illustration of these two PDCM intensity projections on a sample lung are visualized in Fig. 2 (a).

Population-average PDCM angular and radial intensity projections over subjects without emphysema (NE) are displayed in Fig. 2 (b). The averaged angular projection shows a clear pattern of lower attenuations (i.e. intensity values) in the anterior versus posterior region, which agrees with the intensity gradient due to gravity-dependent regional distribution of blood flow and air [29], [30]. The averaged radial projection shows a slight gradient from core to peel regions, which is likely due to the inclusion of voxels belonging to the mediastinal and costal pleura inside the lung mask.

Population-average PDCM intensity projections over subjects with CLE-, PLE-, and PSE-predominant emphysema subtypes are visualized in Fig. 2 (c). To highlight differences with respect to the control set, we display relative values after subtraction of the values from the corresponding NE average projection in Fig. 2 (b). Color coding represents relative intensity differences with more emphysema (more negative attenuation values) corresponding to the red color.

We can see on the relative *angular* PDCM intensity projections that regions of normal attenuation (green to blue) are absent for PLE-predominant subjects, whereas CLE- and PSE-predominant subjects appear to have emphysema regions (red) concentrated in the superior part. The average relative *radial* PDCM intensity projections on emphysema subjects show systematic lower attenuation values consistent with more emphysema in the core part for CLE-predominant subjects and more emphysema in the peel part for PSE-predominant subjects.

### C. Qualitative Evaluation of Discovered sLTPs

For the discovery of sLTPs, 3/4 of the total scans in MESA COPD study (N = 238) were used for training, using random stratified sampling without replacement, while the other scans (N = 79) were used for testing. We summarize the setting of pre-defined parameters for the sLTP learning in TABLE I. In addition, spatial regularization weight *λ* is set via empirical tuning using Eq. (II-D). Based on the relative texture homogeneity loss measure Δ*SSW_T_*, we chose *L*_*T*_ = 1% which corresponds to *λ* = 1.52, above which Δ*SSW*_T_ increases drastically.

**Fig. 3.**
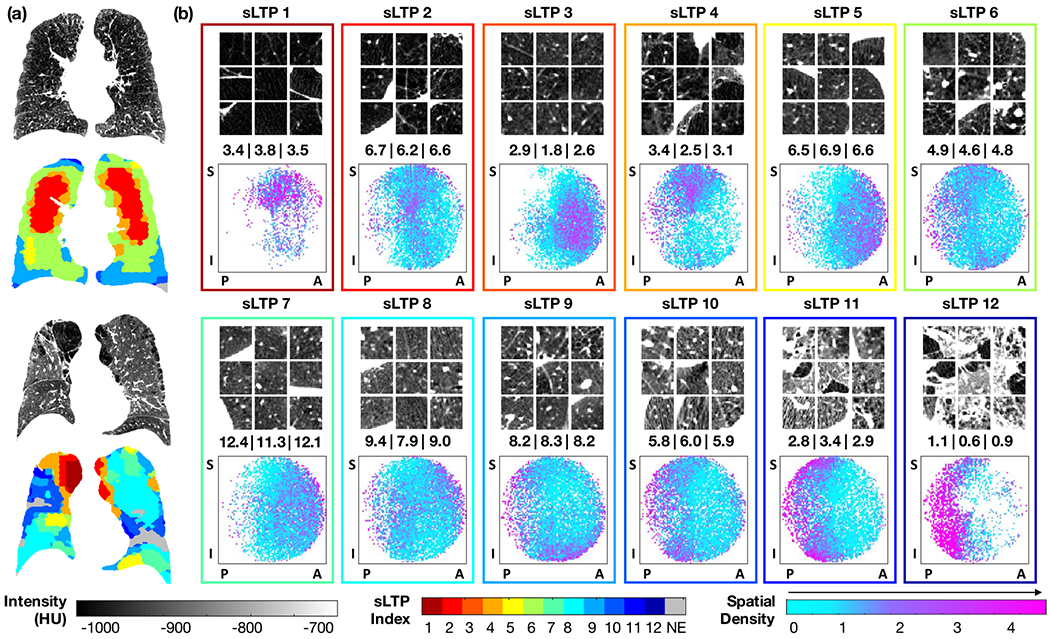
Qualitative illustrations of discovered sLTPs ordered in ascending order of their mean intensity values, equal to: [1: −964, 2: −941, 3: −926, 4: −912, 5: −909, 6: −907, 7: −895, 8: −877, 9: −876, 10: −854, 11: −818, 12: −760] HU. **(a)** Two examples of lung scans and their sLTP labeled masks; **(b)** Characteristics of {*sLTP_k_*}_*k*=1,.., 12_: (top) texture appearance (visualized on axial cuts from 9 random ROIs); (middle) average *%sLTP_k_* on MESA COPD scans with *%sLTP_k_* > 0 within training | test | all cases; (bottom) Spatial density plots of *sLTP_k_* using labeled ROIs (legend: S = superior; I = inferior; P = posterior; A = anterior positions).

A total of 12 sLTPs were discovered using the full training set, and were used to label both the training and test scans in emphysema-like lung. Each sLTP was detected (i.e. *%sLT P*_*k*_ > 0) in at least 5% of scans both in training and test sets. In Fig. 3, we illustrate in (a) the sLTP labeling of two sample CT scans; and in (b) the characteristics of each sLTP via visual illustrations of labeled patches, average occurrence in MESA COPD scans, and spatial distribution of their occurrence within the lungs. For the patch illustrations, 9 samples were randomly selected from all available labeled ROIs (see the Supplementary Material for high-resolution illustrations). For the average occurrence, we averaged *%sLT P*_*k*_ values over scans with *%sLT P*_*k*_ *>* 0. For the spatial distributions, we generated spatial scatter plots of sLTP locations from labeled ROIs, following the method described in II-G, with $N_{\bar{\mathrm{Iso}V}}=5, 000$, and *N*_*r*_ = 60.

We can observe that patches belonging to an individual sLTP appear to be textually homogeneous. sLTP 1 and 4 show clear spatial accumulation in superior (apical) regions, sLTP 3, 5 and 7 in anterior regions, and sLTP 10, 11 and 12 in posterior regions. The brightest LTPs (11 and 12) have very distinct visual appearance and resemble combined pulmonary fibrosis emphysema (CPFE). Since we jointly enforce spatial prevalence and textural homogeneity, some sLTP can have spatial “outliers” that are texturally favored. All sLTPs returned similar occurrences in training and test sets. Some sLTPs are rare, such as sLTP 12 which covers ~1% of the lungs when present, but is still found in 24 scans over the whole MESA COPD cohort.

### D. Reproducibility of sLTPs

#### 1) Reproducibility of sLTP Labeling Versus Training Sets:

To test the reproducibility of sLTPs learning, we first compare the *N*_sLTP_ = 12 sLTPs {*sLT P*_*k*_} generated with the full set of training scans, to *N*_set_ = 4 sLTPs sets ${\left\{sLT\;P_{k}^{c}\right\}}_{\left(c=1,2,3,4\right)}$ using subsets of training data by eliminating via stratified subsampling 25% of the training scans without overlap on the left-out scans. Reproducibility of sLTPs is evaluated on the ROI labeling task, by computing the average overlap of labeled test ROIs with the following metric:

(9) $$R_{\text{ln}}=\frac{1}{N_{\text{set}}\cdot N_{\text{sLTP}}}\sum_{c=1}^{N_{\text{set}}}\sum_{k=1}^{N_{\text{sLTP}}}\frac{\left|{\text{Λ}}_{sLT\;P_{k}}\cap^{\text{​}}{\text{Λ}}_{\pi \left(sLT\;P_{k}^{c}\right)}\right|}{\left|{\text{Λ}}_{sLT\;P_{k}}\right|}$$

where Δ_*sLT P_k_*_ denotes the set of ROIs labeled with *sLT P*_*k*_, and *π*() denotes the permutation operator on the $\left\{sLT\;P_{k}^{c}\right\}$ determined by the Hungarian method [31] for optimal matching between sets {*sLT P*_*k*_} and $\left\{sLT\;P_{k}^{c}\right\}$.

Compared with the *N*_sLTP_ = 12 sLTPs learned on the full training set, we discovered $N_{\mathrm{sLTP}}^{c}=12,12,13$, and 13 sLTPs on training subsets. We obtain an overall labeling reproducibility measure of *R*_ln_ = 0.91 which corresponds to a high reproducibility level.

We then further compute the reproducibility measure, denoted as $R_{\ln}^{\prime }$, among training subsets. The metric is similar to Equation 9, replacing {*sLT P*_*k*_} and $\left\{sLT\;P_{k}^{c}\right\}$ with sLTPs $\left\{sLT\;P_{k}^{c1}\right\}$ and $\left\{sLT\;P_{k}^{c2}\right\}$ (*c*1 ≠ *c*2) learned on different training subsets. We obtain an overall labeling reproducibility measure of $R_{\ln}^{\prime }=0.85$ (standard deviation = 0.07).

To evaluate the contribution of spatial features in sLTP learning, we further generate sets of lung texture patterns using only texture features (i.e. using initial LTPs without spatial augmentation in Section II-D, and setting *λ* = 0 for the replacement test in Section II-E). We discovered 11 patterns using the full training set, and 11, 11, 12 and 12 patterns on training subsets. The reproducibility measures *R*_ln_ and $R_{\ln}^{\prime }$ equal to 0.84 and 0.78 (standard deviation = 0.12), are lower than the ones obtained using the proposed sLTP learning, hence confirming the benefit of adding spatial features.

#### 2) Reproducibility of sLTP Labeling Versus ROI Sampling:

As detailed in Section II-F, sLTP labeling is based on a subset of voxels setting ROI positions, using SURS-based sampling strategy, which is controlled with the parameter *β*_2_ (number of samples per stack). The selected ROIs have an influence on the final outline of the label map, which is hopefully minor if ROIs are sampled densely enough and if sLTPs are generic enough. In this experiment, we test this hypothesis by generating two different sets of ROIs on test scans using two different random seedings, and measure the reproducibility of the generated label masks using the {*sLT P*_*k*_} discovered on the full training set, while varying the *β*_2_ parameter. We measure labeling reproducibility using the two sets of ROIs with the following metrics:

- $R_{\text{1a}}^{DC}\left(sLT\;P_{k},\beta_{2}\right)$ = average of Dice coefficients of label masks of *sLT P*_*k*_ over all test scans;
- $R_{\text{1a}}^{CC}\left(sLT\;P_{k},\beta_{2}\right)$ = Spearman correlation coefficients of *%sLT P*_*k*_ values within the lungs over all test scans.

**Fig. 4.**
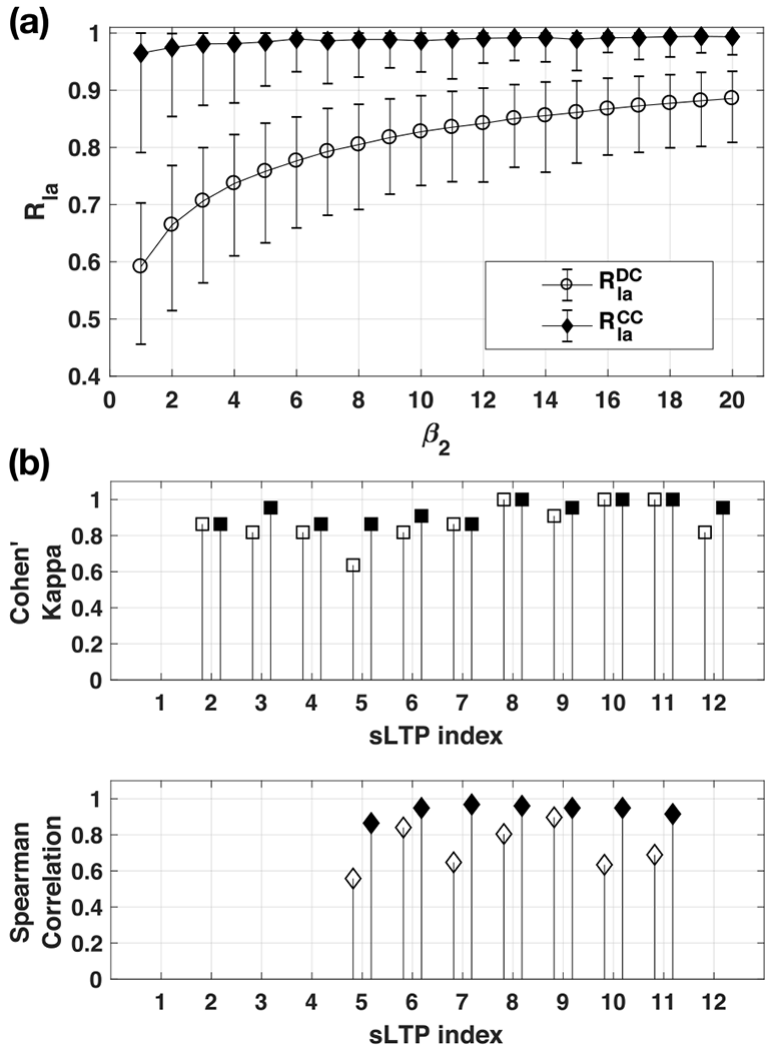
Results of sLTP reproducibility measures. **(a)** Reproducibility measures *R*_la_ versus ROI sampling parameter *β*_2_; **(b)** Reproducibility of sLTPs labeling across scanners (from EMCAP and MESA COPD studies) measured with Cohen’s Kappa coefficients of *sLTP_k_* presence and Spearman correlation coefficients of *%sLTP_k_* values (white=without and black = with intensity histogram mapping).

We illustrate in Fig. 4 (a), the average, max and min values of $R_{\mathrm{la}}^{*}$ measures overall {*sLT P_k_*}, for *β*_2_ ∈ [1, 20]. Both reproducibility measures increase with *β*_2_ in an exponential manner. We obtain an average $R_{\mathrm{la}}^{DC}>0.8$ when *β*_2_ > 10, corresponding to sampling less than 0.05% points in each stack. We obtain an average $R_{\mathrm{la}}^{CC}>0.9$ when *β*_2_ > 5. Minimum *R*_la_ values always occur for sLTP 12, which is the rarest sLTP, as reported in Section III-C.

#### 3) Reproducibility of sLTP Labeling Versus Scanner Type:

The 22 subjects from MESA COPD previously scanned within the EMCAP study, underwent different generations of CT scanners. The average time lapse between EMCAP and MESA COPD scans is 14-months. The mean of *%emph*_−950_, calibrated for outside air values, is 0.7% (min < 0.1%, max = 3.9%) in EMCAP, and 2.6% (min = 0.3%, max = 9.5%) in MESA COPD, corresponding to an average increase of *%emph*_−950_ equal to 1.9%. Therefore, we use this subset of scans to evaluate the reproducibility of sLTP labeling versus scanner types.

We used the 12 sLTPs discovered on the full MESA COPD training set. Because of differences in scanner generations (axial CT in EMCAP versus spiral CT in MESA COPD) and radiation dose settings, intensity calibration was required, implemented in two steps: 1) equalizing the outside air mean intensity value (according to [25]); 2) histogram mapping of normal lung parenchyma identified with the HMMF-based emphysema masks. The sLTPs 2 to 12 were found to be present in both datasets, but sLTPs {2, 3, 4, 12} occur in less than 6 pairs of scans. We report in Fig. 4 (b) the Cohen’s Kappa coefficients of *sLT P*_*k*_ presence for sLTPs 2-12, and the Spearman correlation coefficients of *%sLT P*_*k*_ for the frequent sLTPs only (sLTPs 5 to 11). The Cohen’s Kappa coefficients and Spearman correlations are all above 0.8, which confirms robust sLTP presence and percentage labeling on the 22 subjects scanned on different scanner types in two studies.

### E. sLTPs’ Ability to Encode Standard Emphysema Subtypes

When generating unsupervised lung texture patterns (either sLTPs in this work or earlier generations of LTPs in previous work), we expect them to be finer-grained than the three standard emphysema subtypes used in [4], while still capable to encode them, hence linking unsupervised image-based emphysema subtyping with clinical prior knowledge.

The (s)LTPs (either LTPs or sLTPs) can correspond to a single standard subtype or a mixture of those. We hereby evaluate the ability of the generated (s)LTPs to predict the overall extent of standard emphysema subtypes. To do this, we generate, for each scan and per lung, two signature vectors: 1) a (s)LTP signature histogram composed of the percentage of non-emphysema class (obtained as in Section II-F) and the percentages of individual (s)LTPs in the emphysema-like lung. This normalized histogram is called the (s)LTP predictor signature and is of size $N_{\text{predictor}}=N_{\left(s\right)LTP}+1$; 2) a ground-truth signature composed of the percentage of non-emphysema and the three standard emphysema subtypes (CLE, PLE, PSE), as visually evaluated in [4]. A constrained multivariate regression model is used on labeled training scans to learn regression coefficients between the (s)LTP and ground-truth signatures, using the following optimization:

(10) $${\text{argmin}}_{A}{\left\|XA-Y\right\|}_{2}^{2}\;\mathrm{s.t.}\;0<A_{\left(k,i\right)}<1\;\text{and}\;\sum_{i}A_{k,i}=1$$

where *X*_*N*_scan_ × *N*_predictor__ is composed of all training (s)LTP signatures in *N*_scan_ training scans, and *Y*_*N*_scan_ × 4_ contains the ground-truth signatures. *A*_*N*_predictor_ × 4_ is the matrix of regression coefficients {*A_k,i_*}, which measure the probability of a voxel labeled as a certain predictor belonging to one of the ground-truth classes, and are therefore constrained to be in the range of [0, 1]. Optimization of regression was solved using the CVX toolbox [32]

Quality of prediction is measured with the intraclass correlation (ICC) between predicted and ground-truth exploiting the full MESA COPD dataset. We use a 4-fold cross validation (3/4 label masks used for training the regression and 1/4 used for testing and measuring prediction quality). Significance of differences in ICC values was assessed using Fisher’s r-to-z transformation and a two-tailed test of the resulting z-scores.

**Fig. 5.**
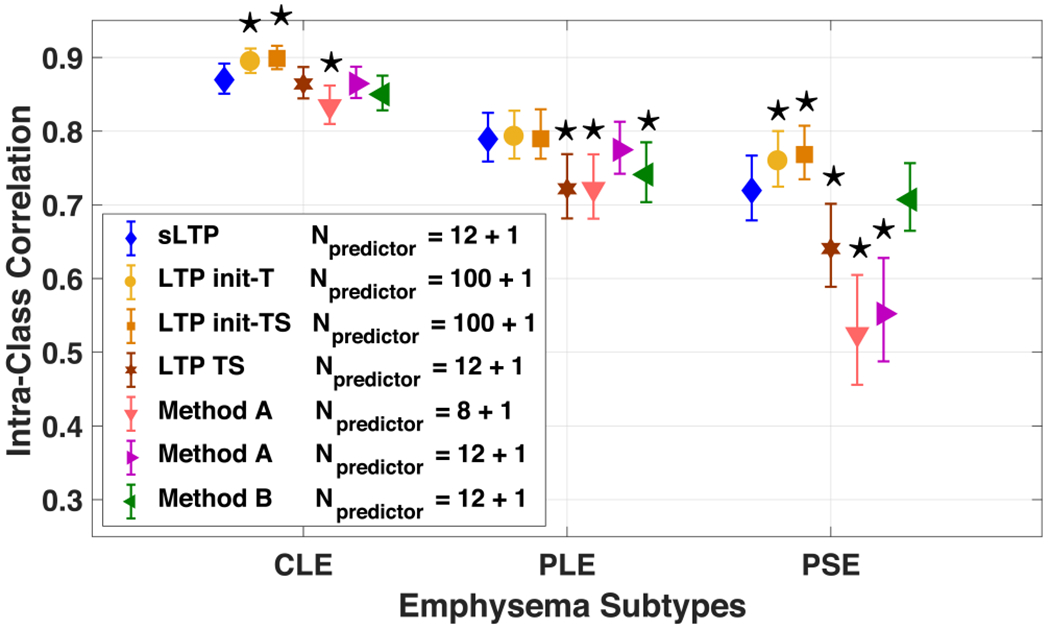
Intraclass correlation (ICC) and 95% confidence interval between predicted standard emphysema subtype scores and ground-truth. Differences with sLTP-based values are marked as ⋆ when significant (*p* < 0.05).

In Fig. 5, we compare prediction quality with 7 sets of *emphysema-specific* (s)LTPs (re)trained on the same set of emphysematous ROIs: 1) the 12 sLTPs learned in this study; 2-3) the initial set of 100 LTPs generated in this study before (denoted as LTP init-T) and after (denoted as LTP init-TS) spatial augmentation; 4) LTPs generated by one-stage clustering (denoted as LTP TS) of the proposed texture and spatial features, by setting *N*_*LT P*_ = 12 directly (this is to test the contribution of the proposed two-stage learning in Section II-D); 5-6) LTPs re-generated using Method A [17], discovered via graph partitioning of 100 candidates based on local spatial co-occurrence and with *N*_*LT P*_ = 8 as in [17] or 12; 7) LTPs re-generated using Method B [18], discovered via merging 100 candidates based on texture similarity and local spatial co-occurrence, and setting *N*_*LT P*_ = 12 for the iterative merging.

Fig. 5 shows that the two sets of 100 LTP models achieve overall best prediction accuracy, and that the newly discovered 12 sLTPs have the best performance among the 5 small (s)LTP sets. Difference of ICC values between the sLTPs and the 100 LTP models was not significant for PLE emphysema subtype.

### F. Clinical Associations of sLTPs

**Fig. 6.**
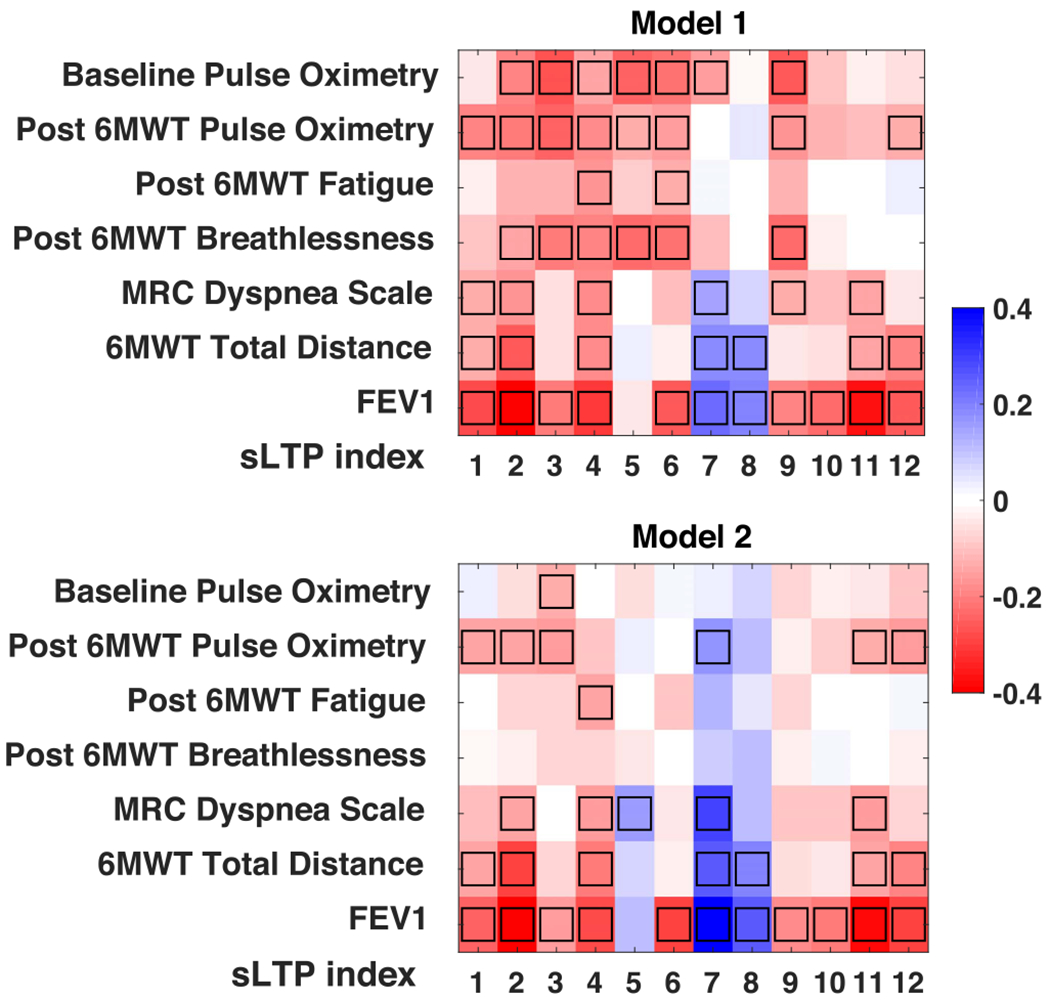
Partial correlations between *%sLTP_k_* and clinical measures after adjusting for demographical factors (Model 1), and adjusting for demographical factors and *%emph*_−950_ (Model 2). Black-boxes indicate statistically significant values (*p* < 0.05).

To evaluate clinical association of sLTPs, we first compute Spearman’s partial correlations between *%sLT P*_*k*_ within both lungs and the seven clinical characteristics listed in III-A, on the full MESA COPD dataset, using two models: Model 1 adjusted for demographical factors (age, race, gender, height and weight), and Model 2 further adjusted for *%emph*_−950_. The results are reported in Fig. 6. Correlation values for MRC dyspnea scale, post 6MWT breathlessness and post 6MWT fatigue are flipped in the figure so that more negative correlation values always correspond to more severe symptoms.

Overall, we obtained 47 and 31 significant correlations with Models 1 and 2. The sLTPs 7 and 8 are associated with less severe symptoms (positive correlations), while the other sLTPs correlate with symptoms (negative correlations). In Model 1, all clinical variables show significant correlations with 2 to 11 sLTPs. Model 2 looses significant correlations for post 6MWT breathlessness, but preserves all, or almost all, significant correlations for FEV1, 6MWT total distance, dyspnea and post-6MWT oximetry. With further adjustment for FEV1 in Model 2, sLTP 3 remains significantly correlated with baseline and post-6MWT pulse oximetry, sLTPs 2, 4 and 7 remain significantly correlated with 6MWT total distance, and sLTP 7 remains significantly correlated with MRC dyspnea scale.

## IV. Discussion & Conclusion

In this work, we propose a novel unsupervised learning framework for discovering *emphysema-specific* lung texture patterns and a small set of emphysema subtype candidates on the MESA COPD cohort of CT scans. The proposed method incorporates spatio-textural features via an original cost metric combining *χ*^2^-*ℓ*^2^ constraints, along with data-driven parameter tuning, and Infomap graph partitioning.

Our methodological framework includes the introduction of a standardized spatial mapping of the lung shape utilizing Poisson distance map and conformal mapping to uniquely encode 3D voxel positions and enable comparison of CT scans. Our PDCM lung shape spatial mapping enables straightforward population-wide study of emphysema spatial patterns. By visualizing relative *angular* PDCM intensity projections on CLE-, PLE- and PSE-predominant subjects, we can see that regions of normal attenuation are absent for PLE-predominant subjects, which agrees with the definition of PLE (diffuse emphysema subtype). CLE- and PSE-predominant subjects appear to have emphysema regions concentrated in the superior part. This agrees with the observation made in [4] on the same dataset that CLE and PSE severity was greater in upper versus lower lung regions, whereas severity of PLE did not vary over the lung. By visualizing relative *radial* PDCM intensity projections, we can see that emphysema subjects show systematic lower attenuation values than subjects without emphysema, as expected. CLE-predominant subjects have more emphysema in the core part, whereas PSE-predominant subjects have more emphysema in the peel part. This agrees with the definitions of CLE and PSE. As a standardized tool, the proposed PDCM spatial mapping is not tied to emphysema patterns, and our future work will exploit such spatial mapping to study other pulmonary diseases.

With the proposed method, we discovered 12 spatially-informed lung texture patterns (sLTPs) in the MESA COPD Study. Qualitative visualization show that the discovered sLTPs appear to be textually homogeneous with specific average intensities and/or spatial prevalence. Using texton-based features to encode both texture and intensity is supported by [33] where “*combination of both texture and densitometric measures strengthened the association with lung function*” as we rely on association with physiological symptoms to evaluate our sLTPs. sLTPs (11, 12) resemble CPFE studied in [34], where posterior emphysematous areas were more likely involved with interstitial lung abnormalities, which agrees with the posterior spatial prevalence seen in Fig. 3.

Extensive evaluations show that the discovered sLTPs are reproducible with respect to training sets, sampling of ROI for labeling, and certain scanner changes. The proposed incorporation of spatial and texture features obtains higher learning reproducibility compared to using texture features only, confirming the benefit of spatial regularization. The number of discovered sLTPs varies slightly between training subsets. This can be caused by a large change in the proportion of rare LTPs within these subsets, which modifies the weights in the Infomap similarity graph. A larger dataset with more diseased cases might be beneficial to solve such issue and would enable us to measure reproducibility on non-overlapping training subsets, which is a limitation of our study.

The sLTPs are able to encode the three standard emphysema subtypes, and thus link unsupervised discovery with clinical prior knowledge. Prediction quality is better than previous models, and close to the optimal level reached with 100 *emphysema-specific* LTPs. While intra-cluster LTP homogeneity increases with the number of LTPs, hence leading to higher prediction performance, working with 100 LTPs leads to redundancy between subtypes which is detrimental when studying associations of individual LTPs with clinical measures. One-stage clustering leads to significantly lower prediction power for PLE and PSE subtypes compared to sLTPs, which demonstrate the benefit of the proposed two-stage learning.

Significant correlations with physiological symptoms were found for several measures. Training our discovery of *emphysema-specific* sLTPs on ROIs with *%emph* > 1 aimed to enable discovery of early emphysema stages. Our correlation results suggest that sLTPs 7 and 8 are good candidates for early emphysema characterization, not yet associated with physiological symptoms. Significant correlation results after adjusting for *%emph*_−950_ indicate that our sLTPs provide clinically-relevant and complementary information to the commonly used *%emph*_−950_ measure. After adjusting for FEV1, there are still sLTPs showing significant correlations with MRC dyspnea scale, 6MWT total distance, baseline and post-6MWT oximetry. Overall, our correlation levels compare well with [35] performed on a similar cohort size, but with highest COPD-prevalence, while reporting fewer significant positive correlations when proposing 7 radiological emphysema subtypes (called “factors”) learned from 80 emphysema visual patterns. Correlations with standard emphysema subtypes, using similar models, were studied for the same population in [4]. Without adjusting for FEV1, CLE and PLE only showed significant associations with MRC dyspnea scale and 6MWT total distance, and only CLE showed significant associations with FEV1. With further adjustment for FEV1, only CLE and PLE showed significant associations with 6MWT total distance.

Progression patterns of the sLTPs will be investigated in the future, via sLTP labeling of longitudinal CT scans (with large time lapse). The sLTP histograms extracted in this study provide texture signatures that can be used to characterize and group CT scans. Patient grouping was found beneficial to study physiological indicators of COPD in [16], and will be considered in our future study. Further development is possible to improve the generation of image-based sLTPs with demographic and population-wide information, which would likely reveal population-specific and population-invariant patterns, but requiring a larger and more diseased cohort for training.

## Supplementary Material

supp1-3094660

## Figures and Tables

**TABLE I:** Parameter Setting for sLTP Learning

Parameters	Setting
ROI size	= 25 mm^3^, to approximate the size of secondary pulmonary lobules
*β*_1_: random shift (for ROI sampling)	∈ [0, 25] mm
*β*_2_: sample density (for ROI sampling)	= 3 samples per stack
# of textons: (for texture features)	= 40, targeting 10 textons per standard emphysema subtype and normal tissue class, according to [13]
Texton size	3×3×3 pixels, according to [18]
# of lung sub-regions (for spatial features)	= 36, according to binning of (*r, θ, ϕ*) in Section II-C3
*N_L T P_*: # of LTPs in initial set	= 100, as suggested in [18], for sufficient diversity of the patterns and being able to discover rare emphysema types
