# Supplementary material for: Novel Subtypes of Pulmonary Emphysema Based on Spatially-Informed Lung Texture Learning: The Multi-Ethnic Study of Atherosclerosis (MESA) COPD Study
Source: IEEE Trans Med Imaging. Author manuscript; Available in PMC 2021 Dec 29. (PMC8715521; doi:10.1109/TMI.2021.3094660)
Supplement: supp1-3094660 [file NIHMS1760785-supplement-supp1-3094660.pdf]

SUPPLEMENTARY MATERIAL

Novel Subtypes of Pulmonary Emphysema
Based on Spatially-Informed Lung Texture Learning: the Multi-Ethnic Study of Atherosclerosis (MESA) COPD Study

Jie Yang, Elsa D. Angelini, Pallavi P. Balte, Eric A. Hoffman,
John H.M. Austin, Benjamin M. Smith, R. Graham Barr, and Andrew F. Laine*

1. Pipeline of the Method and evaluation

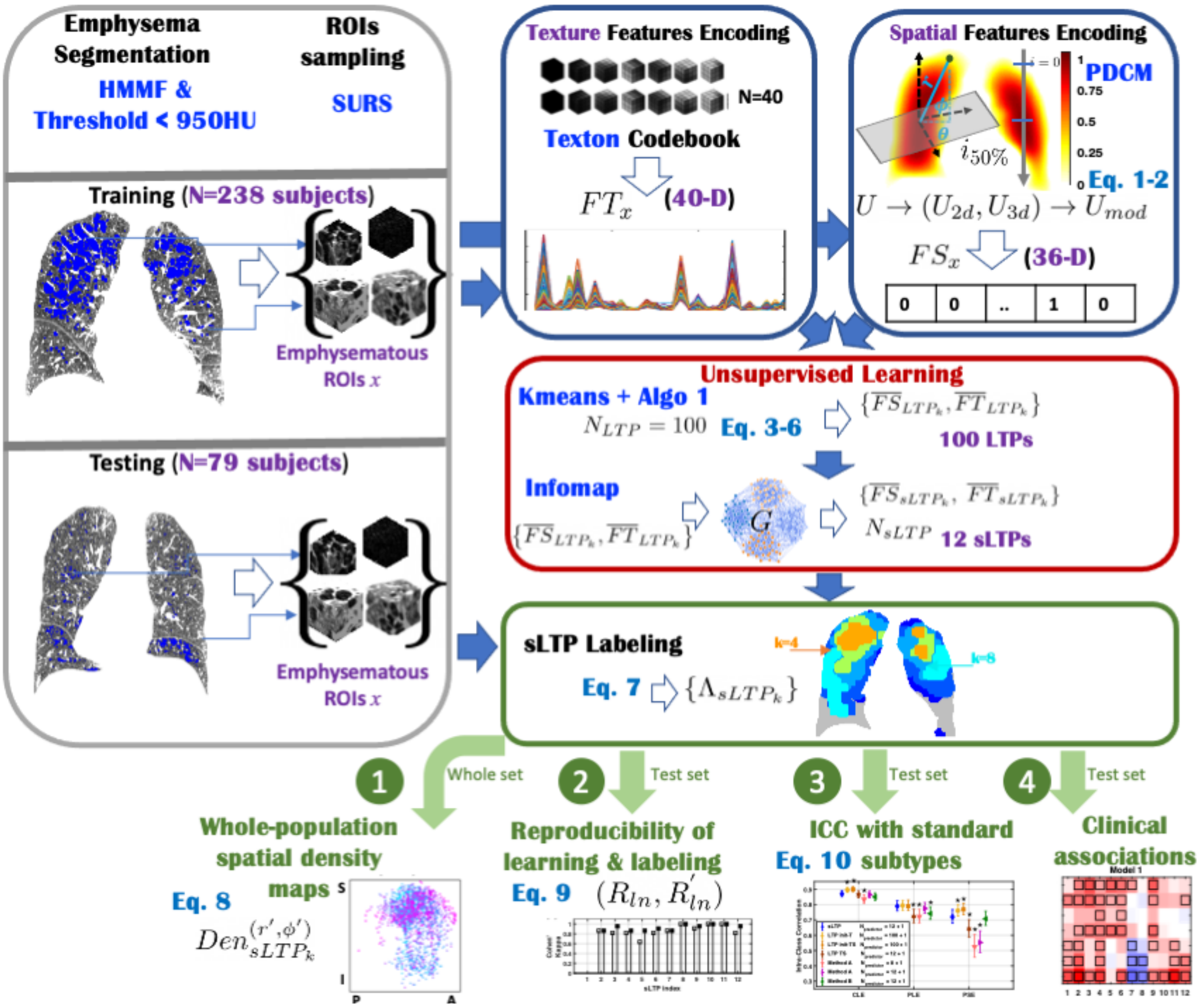

Figure S1: Graphical pipeline of the learning and evaluation steps.

2. High-resolution illustrations of sLTPs

Higher-resolution illustrations of the 12 emphysema subtype candidates called sLTPs (cf. Fig.3 in main manuscript) are provided here on 36 subjects per sLTP, generated as follows:

- Axial ROI size = 73x73 pixels (axial pixel resolutions within the range [0.58, 0.88] mm).
- For each sLTP_k, the 36 subjects with the largest %sLTP_k content are selected, and within a given subject, the axial ROI around the 3D center of the largest sLTP_k connected component is selected. For the rarest sLTPs, the same subject is sampled multiple times, in different connected components.
- Patches are then rescaled to 146 x 146 pixels for higher quality display.
- Display window is [-1000 -700] HU as used in Fig. 3 in the main manuscript.
- sLTPs are ordered according to mean intensity value, similarly to Fig. 3 in the main manuscript.

sLTP #1

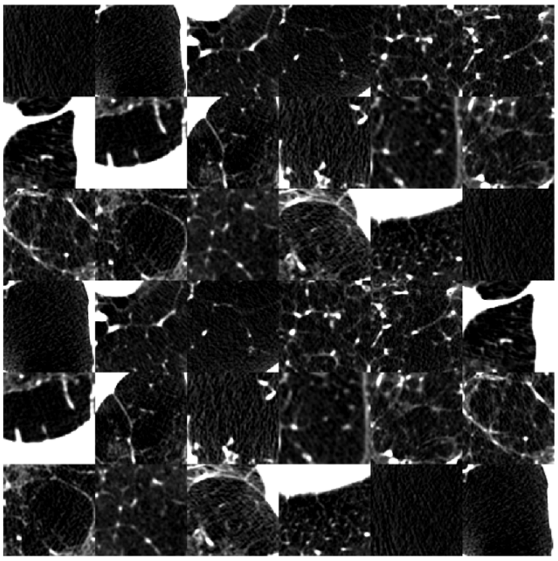

sLTP #2

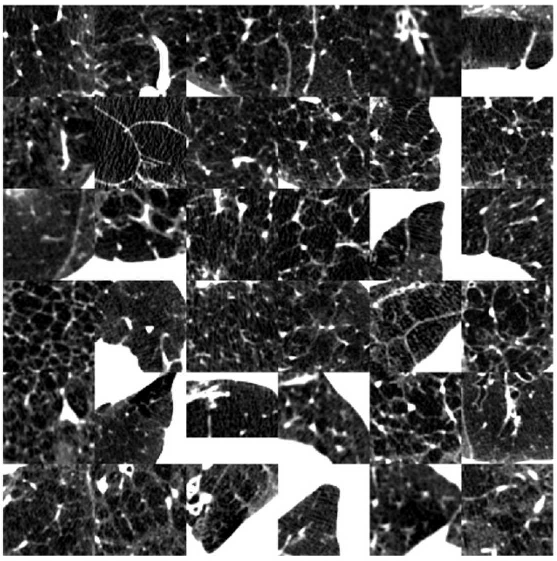

sLTP #3

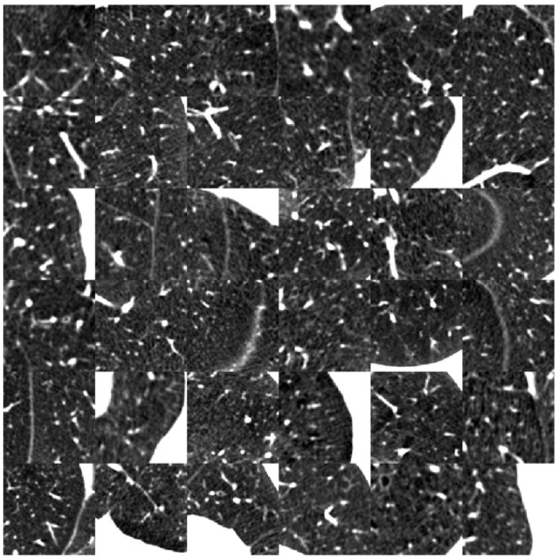

sLTP #4

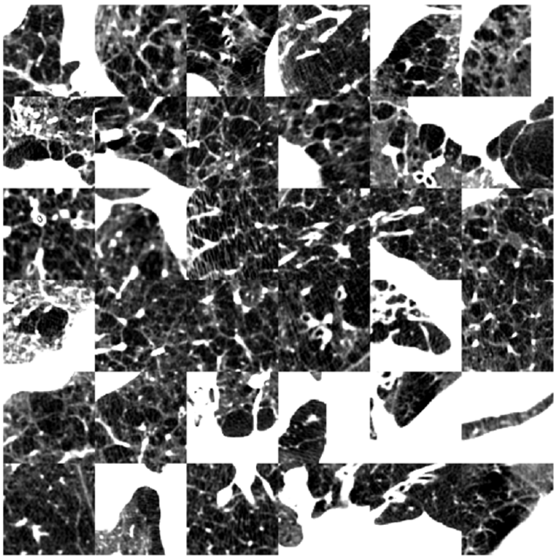

sLTP #5

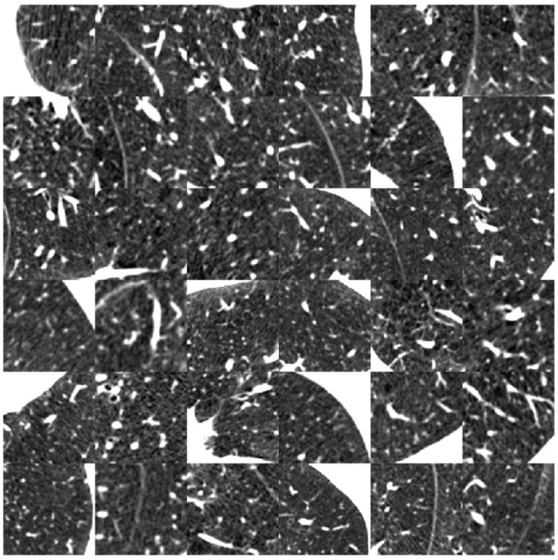

sLTP #6

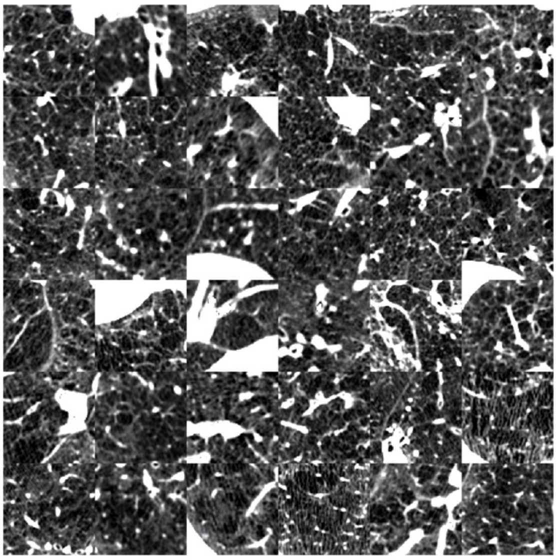

sLTP #7

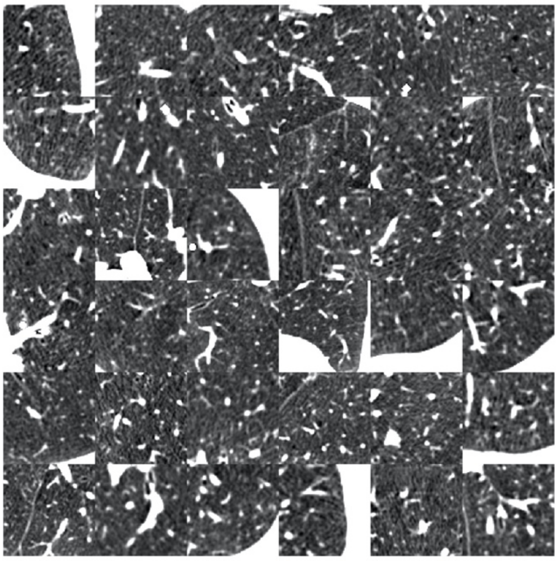

sLTP #8

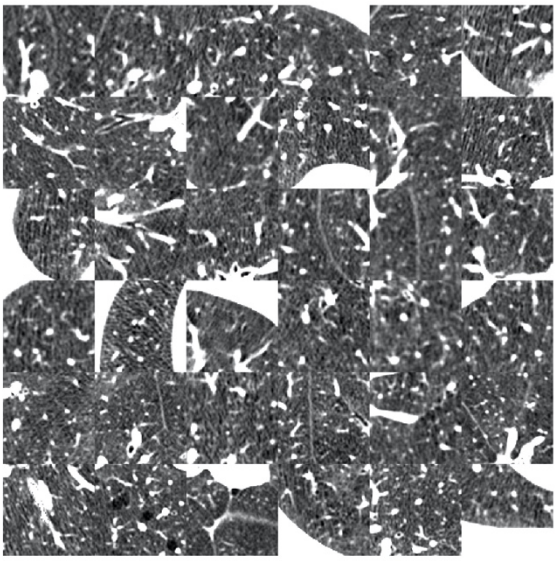

sLTP #9

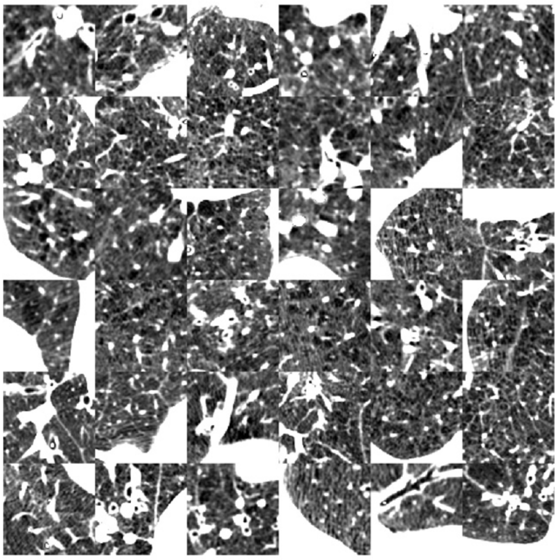

sLTP #10

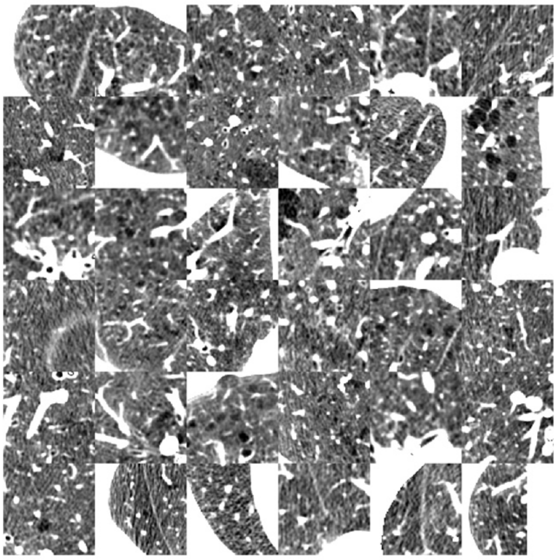

sLTP #11

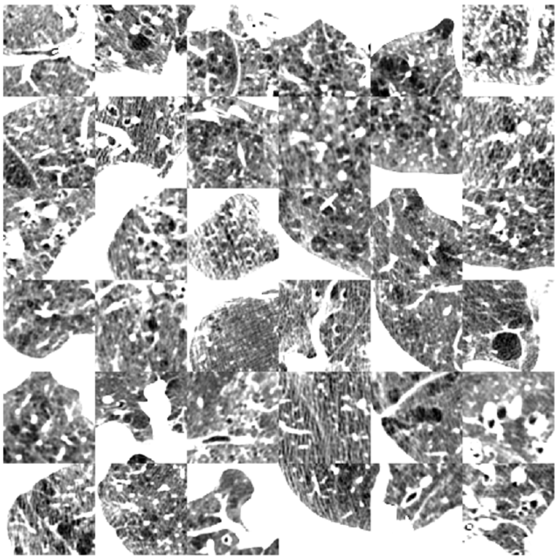

sLTP #12

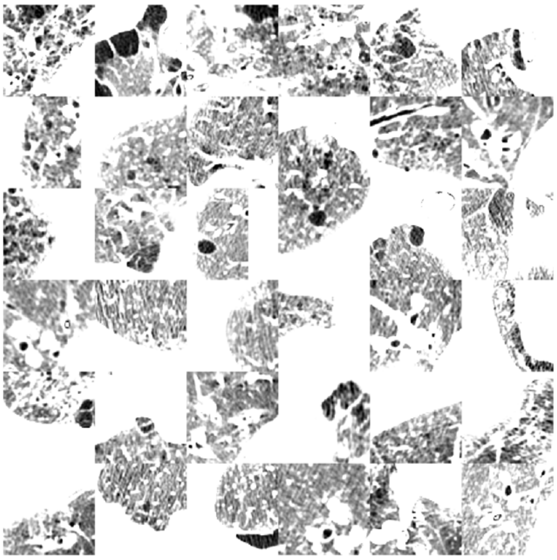

Figure S2: High-resolution illustrations of the 12 emphysema subtype candidates (sLTPs).
